# Apoptosis Induction by *Polygonum minus* Is Related to Antioxidant Capacity, Alterations in Expression of Apoptotic-Related Genes, and S-Phase Cell Cycle Arrest in HepG2 Cell Line

**DOI:** 10.1155/2014/539607

**Published:** 2014-05-13

**Authors:** Mohd Alfazari Mohd Ghazali, Ghanya Al-Naqeb, Kesavanarayanan Krishnan Selvarajan, Mizaton Hazizul Hasan, Aishah Adam

**Affiliations:** ^1^Pharmacology and Toxicology Research Laboratory, Faculty of Pharmacy, University Technology MARA (UiTM), 42300 Bandar Puncak Alam, Selangor Darul Ehsan, Malaysia; ^2^Group on Affinity, Efficacy and Safety Studies (OASES), Brain and Neuroscience Communities of Research, University Technology MARA (UiTM), 40450 Shah Alam, Selangor Darul Ehsan, Malaysia; ^3^School of Pharmaceutical Sciences, University Science of Malaysia (USM), 11800 Minden, Penang, Malaysia; ^4^Department of Food Sciences and Technology, Faculty of Agriculture, University of Sana'a, Sana'a, Yemen

## Abstract

*Polygonum minus* (Polygonaceae) is a medicinal herb distributed throughout eastern Asia. The present study investigated antiproliferative effect of *P. minus* and its possible mechanisms. Four extracts (petroleum ether, methanol, ethyl acetate, and water) were prepared by cold maceration. Extracts were subjected to phytochemical screening, antioxidant, and antiproliferative assays; the most bioactive was fractionated using vacuum liquid chromatography into seven fractions (F1–F7). Antioxidant activity was measured *via* total phenolic content (TPC), 2,2-diphenyl-1-picrylhydrazyl (DPPH), and ferric reducing antioxidant power (FRAP) assays. Antiproliferative activity was evaluated using 3-(4,5-dimethylthiazol-2-yl)-2,5-diphenyltetrazolium bromide (MTT) assay. Most active fraction was tested for apoptosis induction and cell cycle arrest in HepG2 cells using flow cytometry and confocal microscopy. Apoptotic-related gene expression was studied by RT-PCR. Ethyl acetate extract was bioactive in initial assays. Its fraction, F7, exhibited highest antioxidant capacity (TPC; 113.16 ± 6.2 mg GAE/g extract, DPPH; EC_50_: 30.5 ± 3.2 **μ**g/mL, FRAP; 1169 ± 20.3 **μ**mol Fe (II)/mg extract) and selective antiproliferative effect (IC_50_: 25.75 ± 1.5 **μ**g/mL). F7 induced apoptosis in concentration- and time-dependent manner and caused cell cycle arrest at S-phase. Upregulation of proapoptotic genes (*Bax*, *p53*, and *caspase-3*) and downregulation of antiapoptotic gene, *Bcl-2*, were observed. In conclusion, F7 was antiproliferative to HepG2 cells by inducing apoptosis, cell cycle arrest, and *via* antioxidative effects.

## 1. Introduction

Apoptosis plays an important role in cancer development and is a target for enhancing understanding of cancer and in development of anticancer treatment. Apoptosis is a highly regulated process characterized by cleavage of proteins and activation of caspases in viable cells resulting in DNA fragmentation, chromatin condensation, membrane blebbing, and cell shrinkage [1]. This process is essential for homeostatic mechanism to maintain cellular integrity by removing unwanted, redundant, and damaged cells by noninflammatory ways. However, in many cancer cells, apoptosis is dysregulated due to multiple genetic aberrations and cellular stress, conferring resistance to death in these cells which then stay longer in circulation. In the last two decades, extensive studies aimed at improving understanding of intrinsic signaling pathways that control execution of apoptosis in cancer cells were undertaken. These include the use of antiapoptotic proteins and stimulation of proapoptotic proteins as part of treatment strategy for cancer [2]. Carcinogenesis is also related to excessive free radical formation. Many studies have shown that reactive oxygen species (ROS), reactive nitrogen species (RNS), and other metabolism by-products can cause DNA mutation leading to initiation and progression of cancer. Endogenous and exogenous antioxidants can antagonize the promotion phase of carcinogenesis in many types of malignancies through detoxication of these free radicals [3]. Antioxidants from plants with apoptosis-inducing capabilities have drawn a lot of interest in cancer research due to cost effectiveness as they are abundant in nature and supposedly have fewer side effects than synthetic antioxidants. Much work has been conducted on herbs with antioxidant and anticancer effects [4].

*Polygonum minus*, family Polygonaceae, is locally known as “kesum” in Malaysia. It is extensively used in certain types of Malay dishes such as “laksa” or “asam pedas.” It is used traditionally to treat rheumatism, indigestion, and kidney stones and to control hair dandruff. Researchers have linked the pharmacological effects of this plant to its high antioxidant capacity. Aqueous, methanolic, and ethanolic extracts of this plant showed high antioxidant activity which was contributed mostly by its phenolic compounds [5–9]. Fractions from ethanolic and aqueous extract demonstrated gastroprotective effect by inhibiting ulcer lesions in stomach wall of ethanol-induced gastric ulcer in rats [10, 11]. Antimicrobial activities against several strains of bacteria were shown and moderate antiproliferative effect was observed in human cervical carcinoma (HeLa) cell line with IC_50_ of approximately 30 *μ*g/mL with ethanolic extract [12]. No cytotoxicity was seen in normal human lung fibroblast cell line (Hs888Lu) that was exposed to aqueous and ethanolic extracts [13]. Determination of acute and subacute oral toxicities of aqueous extract in Wistar rats at the highest dose gave no significant changes in food and water intake, behavior, blood chemistry, hematology parameters, and neurological assessment [14].

The present study was undertaken to assess the antiproliferative activity and antioxidant capacity of* P. minus* and to examine mechanism(s) of action of the most active fraction. This involved identification of the most bioactive crude extract in terms of high antioxidant activity and potent antiproliferative activity. This crude extract was further subjected to chromatographic fractionation and retested. The fraction with smallest IC_50_ in antiproliferative assay using HepG2 cells was assessed for apoptosis induction by looking at cell cycle arrest and expression of several apoptotic-related genes.

## 2. Materials

### 2.1. Chemicals

Petroleum ether, methanol, hexane, and ethyl acetate were purchased from Fisher Scientific, USA. Silica gel 60 PF_254_ was procured from Merck, Germany. Folin-Ciocalteu reagent, 2,2-diphenyl-1-picrylhydrazyl, MTT powder, 2,4,6-tris(2-pyridyl)-s-triazine, sodium carbonate, copper sulfate, sodium chloride, sodium potassium tartrate, phosphate buffered saline pH 7.4 (PBS), and gallic acid were acquired from Sigma, USA. Annexin-V and propidium iodide (PI) were obtained from Becton Dickinson, USA. Total RNA Isolation kit and TUNEL assay kit were bought from Promega, USA. All primers were synthesized by Beacon designer, Premier Biosoft International.

### 2.2. Plant Material

Plant material was procured in Seri Kembangan, Selangor, Malaysia. Plant was identified by Dr Shamsul Khamis, Institute of Bioscience, University Putra Malaysia, and a voucher specimen SK 2105/12 was deposited at the herbarium of Atta-ur-Rahman Research Institute of Natural Products (AURiND UiTM).

## 3. Methods

### 3.1. Study Design

**Figure 1:**
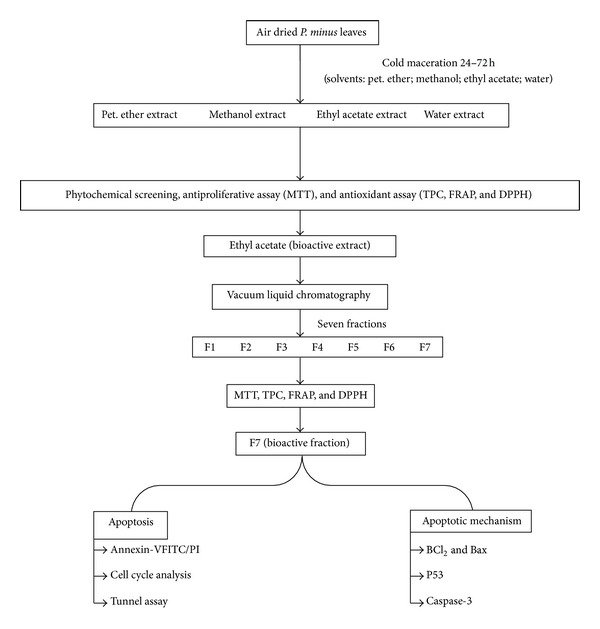
Flowchart of study.

The flow chart of the study is shown in Figure 1.

### 3.2. Extraction and Fractionation

Leaves were manually picked from stems. Fresh leaves of* P. minus* were dried at room temperature for 24 h and subjected to 40°C oven for a week to dry completely. Dried leaves were chopped finely into powder form using a commercial grinder for 15 min. Plant powder was soaked in several organic solvents petroleum ether, methanol (MeOH), ethyl acetate (EtOAc), and water for 24 up to 72 h in a ratio of 1 : 20 w/v as per methods described earlier [15]. The extract was filtered using filter paper Whatman number 1 before being dried at reduced pressure in rotary evaporator (Büchi, Switzerland) to give a green colored extract. Vacuum liquid chromatography (VLC) method was employed to separate the most bioactive crude extract. Silica gel 60 PF_254_ was packed into the VLC column (10 cm × 10 cm) and washed with hexane until fully packed. The crude extract (10 g) was dissolved in ethyl acetate and preabsorbed onto silica gel 60. The column was then eluted* via* gradient elution with a combination of hexane and ethyl acetate in a ratio 9.5 : 0.5. The yield was collected into small bottles and pooled based on thin layer chromatography (TLC) pattern.

### 3.3. Phytochemical Screening

Extract (20 mg) was dissolved in 10 mL of MeOH and filtered. Filtrate (1 mL) was transferred into two test tubes and a few drops of concentrated HCl were added. To test for flavonoids, the first tube was shaken for 2 min, and a piece of magnesium powder was then added. A positive result was indicated by a change in color of the sample to red. The second tube was shaken for 2 min and a few drops of Mayer's reagent were added. Formation of a yellow-cream precipitate showed presence of alkaloids. To detect presence of saponins in extract, 500 mg of extract was mixed with 5 mL distilled water (dH_2_O) in a test tube which was then shaken vigorously. Formation of a stable froth for at least 15 min indicated presence of saponins.

### 3.4. Determination of Antioxidant Capacity

#### 3.4.1. Total Phenolic Content Assay

Total phenolic content (TPC) was measured using procedures described earlier [16]. Gallic acid was used as standard against which TPC of every fraction was compared. Sodium carbonate, copper sulfate, and sodium potassium tartrate in the ratio of 100 : 1 : 1 was mixed well and prepared fresh before experiment. The mixture was pipetted into tubes containing test sample or standard and left for 15 min. After this time, a mixture of Folin-Ciocalteu reagent and water (1 : 3) was added whilst vortexing. The mixture was allowed to stand at room temperature for 35 min in the dark. Absorbance was read spectrophotometrically at 750 nm.

#### 3.4.2. DPPH Scavenging Assay

Principle of this assay is to detect ability of test sample to reduce DPPH free radicals. Method of [17] was used. DPPH was dissolved in ethanol 50% and mixed with HEPES buffer and 0.9% NaCl in 50 mL dH_2_O and adjusted to pH 7.4. Test sample was dissolved in MeOH. Sample (5 *μ*L) was added to 195 *μ*L of ice cold DPPH solution and allowed to stand at room temperature for 30 min. Test sample was then transferred to clear and transparent 96-well plates and was read using a microplate reader (Sunrise, Tecan, Switzerland) at 517 nm. Percent scavenging effect was calculated using the formula:

(1) $$\begin{array}{c} \%\;\text{scavenging}\;\text{effect}=\left(\text{Ac}-\text{As}/\text{Ac}\right)\times 100, \end{array}$$

where Ac is absorbance of control and As is absorbance of sample.

#### 3.4.3. Ferric Reducing Antioxidant Power (FRAP) Assay

Antioxidant activity was measured using a modified FRAP assay method [18]. FRAP values were obtained by comparing the absorbance change at 593 nm in test reaction mixtures with those containing ferrous ions in known concentration. Sodium acetate (300 mM), pH 3.6 in 1 L dH_2_O, 10 mM of 2,4,6-tris(2-pyridyl)-s-triazine (TPTZ) in 100 mL of 40 mM HCL, and 20 mM ferric chloride were combined in a ratio of 10 : 1 : 1 (v/v/v) fresh prior to experiment. Test samples (100 *μ*L) were mixed with 3 mL of FRAP reagent and allowed to stand at room temperature for 4 min before reading the absorbance spectrophotometrically at 593 nm. The FRAP value was expressed as *μ*mol Fe (II)/mg dry weight of extract. FeSO_4_ (2 mM) was used to construct a standard curve.

### 3.5. Antiproliferative Activity

#### 3.5.1. MTT Assay

MTT assay was performed as previously described [19]. Crude extract was dissolved in 100% dimethyl sulfoxide (DMSO) and prepared fresh prior to assay. Cells (2 × 10^4^/well) were plated in 96-well plate and incubated at 37°C for 24 h. Next day, cells were treated with the crude extract in DMSO (<0.5%) and the plate was incubated for 72 h. Then, MTT solution (50 *μ*L) was added into each well and incubated for 4 h. Supernatant in each well was then collected and DMSO (100 *μ*L) was used to dissolve the formazan crystal in the well. The plate was read with a microplate reader at 570 nm.

### 3.6. Flow Cytometry Analysis

Flow cytometry analysis was carried out to investigate proportion of cancer cells undergoing apoptosis using annexin-V conjugated with fluorescein isothiocyanate (FITC) detection kit according to manufacturer's instructions. Cells (1 × 10^6^/well) were seeded onto 24-well plates and incubated at 37°C overnight. Next day, cells were treated with various concentrations of the extract and incubated for 24, 48, and 72 h, respectively. After this, cells were detached using trypsin for 5 min. Cells were collected and centrifuged at 1000 rpm for 5 min. Cell pellet was washed with PBS and resuspended in 100 *μ*L binding buffer. Subsequently, 2.5 *μ*L annexin V-FITC and 3 *μ*L propidium iodide (PI) were added and kept in the dark at room temperature for 15 min. Annexin V-FITC/PI stained cells were analyzed using flow cytometry (FACSCalibur, Becton Dickinson, USA).

### 3.7. Cell Cycle Analysis

Cells were treated as described in flow cytometry analysis. After treatment and incubation for 24 and 48 h, cells were harvested and fixed with ice-cold 70% ethanol (1 mL) at −20°C for 2 h. Ethanol was then removed (1000 rpm, 5 min) and the cells were washed twice with cold PBS. Subsequently, cells were resuspended in 425 *μ*L of PBS, 25 *μ*L PI (1 mg/mL), and 50 *μ*L RNaseA (1 mg/mL) and incubated for 30 min at room temperature. Distribution of the cell cycle was measured by flow cytometer and data analysis was carried out with ModFitLT software (version 4) [20].

### 3.8. TUNEL Assay

TUNEL assay was performed as per procedures described in the kit (DeadEnd Fluorometric TUNEL System kit, Promega, USA). HepG2 cells (0.5 × 10^6^/well) were plated in fluorometric dish overnight. The next day, cells were treated with F7 and incubated for 48 h. Staining protocol was carried out according to manufacturer's instructions of the kit. Specimens were viewed under fluorescence confocal microscope (Leica TCS SPE, Germany) to identify apoptotic cells.

### 3.9. Gene Expression Studies

HepG2 cells were seeded in T25 flasks and allowed to attain 70–80% confluency. The next day, cells were treated with F7 for 48 h using three concentrations (12.5, 25, and 37.5 *μ*g/mL). These concentrations were determined from preliminary experiments to determine growth-response curves by MTT assay and verified by flow cytometry. They correspond to IC_25_,  IC_50_, and  IC_75_, respectively. Total RNA extraction was carried out using the procedures described in Total RNA Isolation kit (Promega, USA). Concentration of RNA was calculated using biophotometer (BioRad, USA) and purity of RNA was determined. Mastermix (12.5 *μ*L) containing SYBR green dye as detector was added with 1.5 *μ*L of pure RNA, 0.5 *μ*L RNase, 1 *μ*L of each forward and reverse primers, and 8.5 *μ*L of dH_2_O to give a final reaction mixture of approximately 25 *μ*L. PCR product was then subjected to Corbett thermal cycler (Corbett Research, Australia). Optimized PCR amplification comprised an initial denaturation step at 42°C for 30 min, followed by denaturation at 95°C for 10 min, annealing at 95°C for 15 sec, extension at 50°C for 30 sec, and final extension step at 72°C for 3 sec. Melt curves were analysed to check for absence of mispriming. Possibility of genomic DNA influence on the results was eliminated by use of primers. Each experiment was performed three times and all samples were run in triplicates. Expression levels for each gene relative to housekeeping gene, *β*-actin, were calculated for all samples using Rotor-Gene (version 1.7, Corbett Research) and Microsoft Excel. Analysis of gene expression data was carried out by ΔΔCT method of relative quantification as described in [21]. Ratio in untreated cells (negative control) was assigned as 1.

### 3.10. Statistical Analysis

Each experiment was conducted three times and determinations were in triplicates. Data were analyzed by analysis of variance (ANOVA) and Bonferroni test using SPSS software (version 20). Statistical significance was fixed at *P* < 0.05. Values are shown as mean ± standard deviation (S.D.).

## 4. Results and Discussion

### 4.1. Extraction and Fractionation

The yield for petroleum ether extract was 12.9 g (10.32%), 12 g (9.6%) for MeOH extract, and 9.8 g (7.84%) for ethyl acetate extract from 125 g of leaf powder. Fractionation of the ethyl acetate extract (10 g) yielded F7 as the highest yield of 2.2 g (22%), F6 of 1.8 g (18%), F5, and F4: 1.2 g (12%) each, F3, F2, and F1, 0.88 g (8.8%), 0.7 g (7%), and 6.8 g (6.8%), respectively.

### 4.2. Antioxidant Activity

The antioxidant activity of crude extracts is shown in Table 1 and fractions isolated from EtOAc crude extract are shown in Table 2.

### 4.3. Phytochemical Screening

TPC of the four crude extracts was determined from a linear gallic acid standard curve (*y* = 0.005*x* + 0.132,  *R* ^2^ = 0.982) (Table 1). There was a significant difference (*P* < 0.05) in TPC among the extracts with EtOAc extract exhibiting the highest TPC. This indicates that EtOAc was the best solvent to extract phenolic components from this plant. Similar findings were reported by [22–24]. Qualitative analysis of* P. minus* crude extracts also pointed to high amounts of flavonoids, a polyphenol group, in EtOAc extract (Table 3). Subsequent fractionation of EtOAc crude extract yielded 7 fractions (Table 2). Of these, F7 had the highest TPC value, higher even than that of Trolox, a vitamin E analogue. Fractionation using solvent elution from nonpolar solvent (hexane) to more polar solvent (EtOAc) yielded fractions with different chemical constituents. TPC content of each of these fractions was significantly smaller than that of crude extract suggesting that the phenolic components were separated based on their polarity. Sum of TPC of these fractions was larger than that of EtOAc crude extract pointing to possible interactions between chemicals in crude extracts which resulted in attenuation of its reducing power which was the main principle of the TPC assay [16].

DPPH assay results were presented as concentration of extract which scavenged 50% of DPPH radicals (EC_50_). EtOAc extract showed lowest EC_50_ of 120.3 ± 2.7 *μ*g/mL in DPPH assay (Table 1).  MeOH and water crude extracts showed large EC_50_ values while petroleum ether extract showed the weakest DPPH scavenging activity. Of the fractions, EC_50_ for DPPH scavenging by F7 was the lowest at 30.5 ± 3.2 *μ*g/mL (Table 2). EC_50_ for F7 was only 1.5 times that of chlorogenic acid which had an EC_50_ of 20 ± 2.8 *μ*g/mL. Results from DPPH assay showed scavenging activity of the fractions in descending order as F7 > F6 > F5 > F4 > F3 > F2 > F1. DPPH assay results correlated well with TPC (*r* = 0.904,  *P* < 0.01). Purification and concentration of polyphenolic compounds must have occurred with increase in polarity of extraction solvents that were used during the fractionation procedures as previously reported [25, 26].

FRAP assay determines the reducing power of test compounds. The assay depends on reduction of ferric tripyridyltriazine complex (Fe (III)-TPTZ) to ferrous tripyridyltriazine (Fe (II)-TPTZ) by a reductant at low pH. FRAP value for EtOAc extract was the highest amongst the crude extracts at 2435.21 ± 28.9 *μ*mol Fe (II)/mg dry weight extract (Table 1).  After fractionation, FRAP values of F1 to F7 were in the range of 147.53 ± 8.4 to 1169.1 ± 20.3 *μ*mol Fe (II)/mg dry weight extract (Table 2). Sum of FRAP values of F1 to F7 was 2356 *μ*mol Fe (II)/mg dry weight extract. This was not much different from FRAP value of crude EtOAc extract. Each of fractions 1 to 7 possibly contributed to total antioxidant capacity shown by the crude EtOAc extract. Tripathi et al. showed that antioxidant compounds in an extract show synergistic effect in their investigations on effect of crude or total extract and their fractions in inflammation iNOS signaling cascade [27]. FRAP values of* P. minus* fractions were linearly correlated to TPC (FRAP versus TPC, *r* = 0.83, *P* < 0.05). Since EtOAc typically extracts polyphenolic compounds [28], F7 most probably contains polyphenols with high antioxidant capacity since polyphenols act as electron donors to stabilize free radicals [29]. Plant extracts that contained high concentrations of phenolics demonstrate high antioxidant effects* in vitro *[30]. Vitamin E, tocotrienols, tocopherols, and ascorbic acid may also contribute to antioxidant capacity as well as to the synergistic effect among the compounds [31]. EtOAc crude extract by qualitative analysis showed presence of high amounts of flavonoids which would act as reductants in TPC and FRAP assays and as scavengers in DPPH assay. Since saponins do not contribute to FRAP values [32], water extract of* P. minus* which showed high content of saponins in qualitative assay had low FRAP value when compared to EtOAc extract.

### 4.4. Antiproliferative Activity

Antiproliferative effects of* P. minus* crude extracts on selected cancer and normal cell lines were determined (Table 4). Of the 4 crude extracts, EtOAc extract had the lowest IC_50_ of 32.25 ± 3.72 *μ*g/mL towards HepG2 with little cytotoxicity towards normal embryonic liver cells (WRL68) or normal Chang liver cells. IC_50_ values of EtOAc crude extract towards these latter two normal liver cell lines were 122.38 ± 20.84 and 285.72 ± 59.8 *μ*g/mL, respectively. Colon cancer cells (HCT 116) and mammary cancer cells (MCF-7) were not much affected by EtOAc extract although there were moderate effects against cervical cancer cell line (HeLA). MeOH extract of* P. minus* also showed moderate antiproliferative effects against HCT 116 although it was ineffective against the other cell lines. Petroleum ether extract was cytotoxic in WRL68 with IC_50_ of 56.23 ± 3.2 *μ*g/mL but has no antiproliferative effects in cancer cell lines. Crude water extract had no antiproliferative effect on cancer cell lines and no cytotoxicity in normal cell lines (IC_50_ > 250 *μ*g/mL in all cases). This finding of water extract being noncytotoxic is important as it provides evidence that the herb is safe when added to certain Malay foods during their preparation.

Our data show that EtOAc crude extract has selective antiproliferative effect on HepG2 with little cytotoxicity on normal liver cells. This is important as selectivity of extract towards cancer cells without effect on normal cells is crucial to ensure that nontarget cells are not affected by treatment so as to gain optimal therapeutic effects with minimal adverse effects. Similar views are abundant in the literature [33, 34]. The bioactive compounds in EtOAc crude extract inhibited proliferation of HepG2 cells. This extract was thus fractionated for further biological investigations on* P. minus*. Fractions of EtOAc extract displayed better antiproliferative activity than the crude extract (Table 5). F7 exhibited the smallest IC_50_ of 25.75 ± 1.5 *μ*g/mL towards HepG2 cells compared to the other fractions. F7 also showed the highest antioxidant capacity in TPC, FRAP, and DPPH assays. Qualitative analysis of EtOAc crude extract from which F7 was obtained showed it to be high in flavonoids, alkaloids, and saponins; these groups of compounds most probably contributed to antiproliferative effects of F7.  In many reports, flavonoids were shown to selectively kill cancer cells which frequently have higher levels of ROS than normal cells [35]. Consistently elevated levels of ROS activate an adaptive stress response which enables cancer cells to survive in high ROS ambience to maintain cellular viability [36]. Flavonoids are known as effective scavengers of ROS and are able to modulate proteins that are involved in cell proliferation mainly through regulation of the cell cycle [37]. In addition, flavonoids were able to induce apoptotic death in many cancer cells and act as antiangiogenic agents [38]. As example, luteolin, a common flavonoid in plants, was found to arrest the cell cycle at G_1_ phase in human gastric, prostate, and melanoma cancer cells and was an apoptotic inducer at both intrinsic and extrinsic pathways. Luteolin exhibited antiangiogenesis effect by suppressing vascular endothelial growth factor and matrix metalloproteases and was antimetastasis through inhibition of tumor necrosis factor and interleukin-6 which regulate cell migration and metastasis [39]. Besides exhibiting similar mechanisms of action as flavonoids, alkaloids and saponins may also contribute to antiproliferative effects by inducing cell cancer autophagy and antimultidrug resistance and antimitotic effect [40]. Existing data on concomitant use of antioxidant compounds and chemotherapy showed that antioxidant supplementation led to improvement in treatment outcomes, increased survival times, increased tumor response, and reduced toxicity [41]. However, some studies also showed no significant improvements in treatment outcomes that could be attributed to use of insufficient antioxidant doses [42]. Chemotherapeutic drugs like doxorubicin and vinorelbine work* via* mechanisms that involve induction of apoptosis in cancer cells through ROS-mediated oxidative stress [43, 44]. For these types of drugs, concomitant supplementation with antioxidants during cancer chemotherapy may lead to diminished anticancer effects. However, no trials have reported significant reductions in treatment efficacy with antioxidant supplementation, suggesting no evidence of antioxidants interfering with chemotherapy [41, 45].

### 4.5. Apoptosis Induction by EtOAc Extract and F7 of* P. minus* in HepG2 Cells

**Figure 2:**
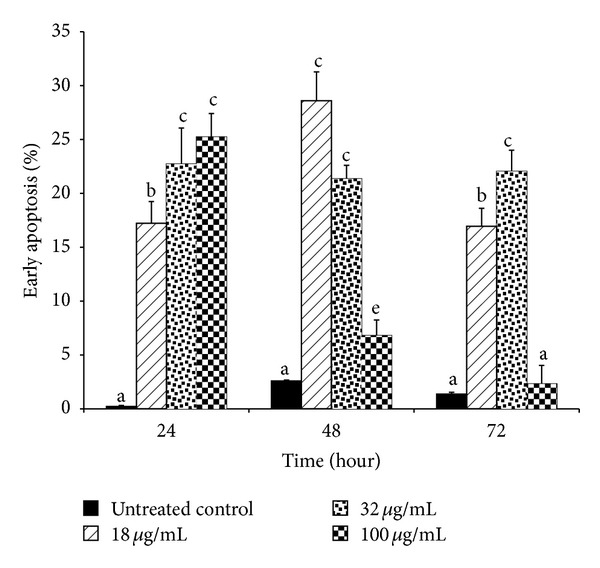
Early apoptosis in HepG2 cells exposed to EtOAc crude extract of* P. minus*. HepG2 cells (1 × 10^6^/well) were incubated with EtOAc crude extract of* P. minus* (18–100 *μ*g/mL) for different periods (24, 48, and 72 h) at 37°C. Apoptosis was detected by flow cytometry using annexin-V and FITC. Results are mean ± S.D. (*n* = 3). Values with different alphabets are significantly different (*P* < 0.05, ANOVA and Bonferroni test).

**Figure 3:**
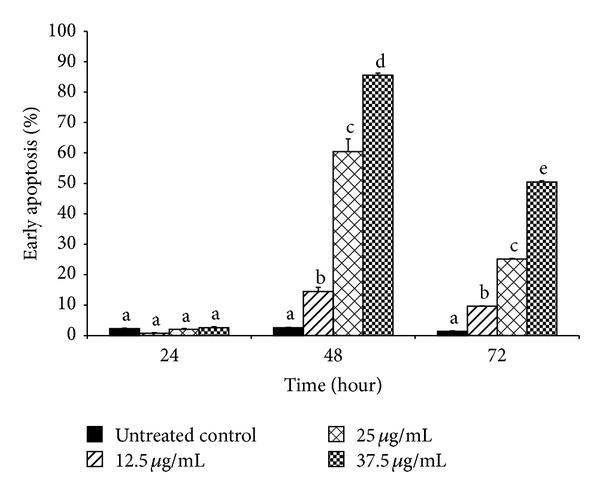
Percentage of HepG2 cells in early apoptosis upon exposure to F7 of* P. minus*. HepG2 cells (1 × 10^6^/well) were incubated with F7 of* P. minus* (12.5–37.5 *μ*g/mL) for different periods (24, 48, and 72 h) at 37°C. Apoptosis was detected by flow cytometry using annexin V-FITC. Results are mean ± S.D (*n* = 3). Values with different alphabets are significantly different (*P* < 0.05, ANOVA and Bonferroni test).

**Figure 4:**
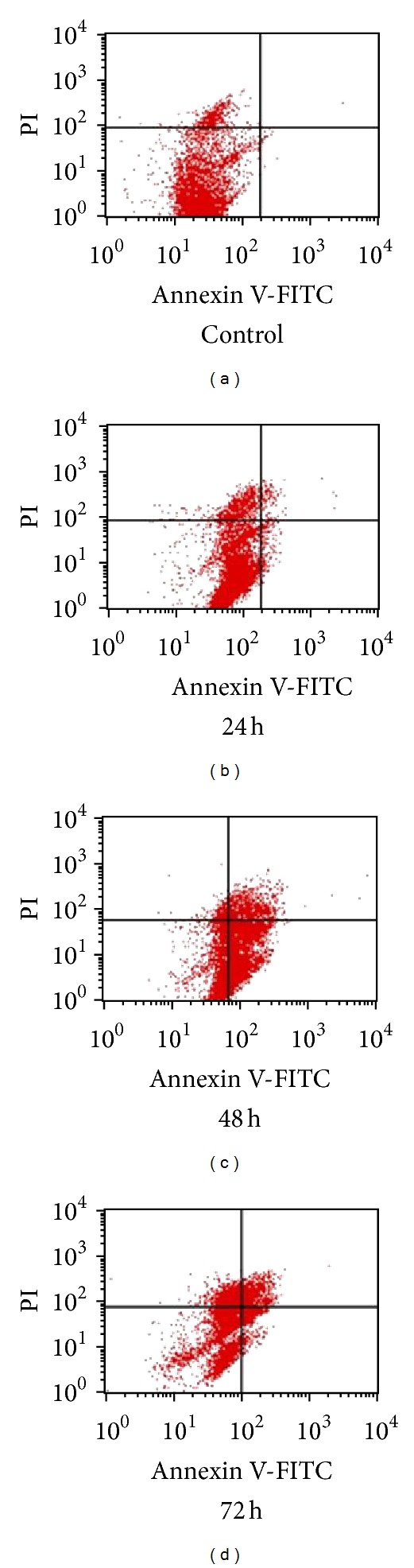
Histogram of annexin-V and FITC/PI flow cytometry of HepG2 cells exposed to F7. HepG2 cells (1 × 10^6^/well) were incubated with F7 of* P. minus* (25 *μ*g/mL, equivalent to IC_50_) or (a) control for (b) 24, (c) 48 and (d) 72 h at 37°C. Lower left quadrant in each panel represents viable cells which excluded PI and were negative for annexin V-FITC binding. Upper right quadrant contains nonviable, necrotic cells or late stage apoptotic cells, positive for annexin V-FITC/PI uptake. Lower right quadrant contains early apoptotic cells, annexin V-FITC positive and PI negative. One experiment is representative of three independent experiments.

**Figure 5:**
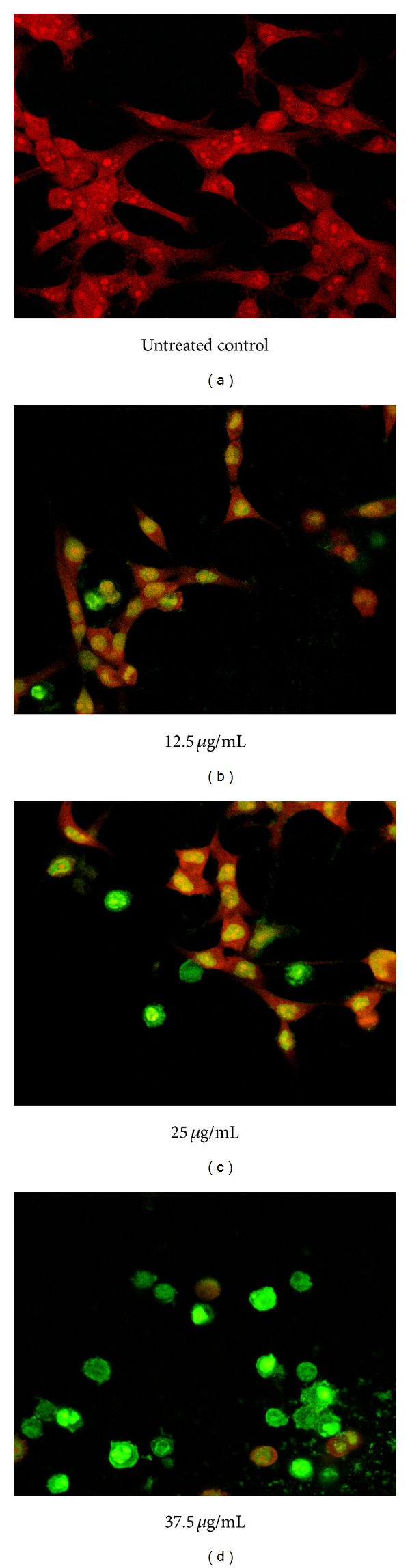
Fluorescence confocal imaging of TUNEL positive staining in HepG2 cells exposed to F7 at 48 h. HepG2 cells (0.5 × 10^6^/well) were treated with F7 of* P. minus* (12.5–37.5 *μ*g/mL) for 48 h at 37°C. Red stain shows viable, healthy cells while intense green stain indicates apoptotic cell with DNA fragmentation. One experiment is representative of three independent experiments.

Mode of cell death induced by F7 was examined* via* detection of translocation of phosphatidylserine into outer cell membrane. This is a hallmark event for early apoptosis whereby cancer cells will emit the signal to surrounding macrophages to initiate autophagy [46]. EtOAc crude extract elicited a significant increase in percentage of HepG2 cells in early apoptosis (Figure 2). Lowest concentration of EtOAc crude extract (18 *μ*g/mL) showed time-dependent increase in percentage of cells undergoing early apoptosis. Exposure to 24 h produced about 20% increase. After 48 h of exposure, maximal increase to nearly 30% was seen, but after 72 h, percentage of cells in early apoptosis had dropped significantly to less than 20%. A higher concentration (32 *μ*g/mL) of EtOAc crude extract increased percentage of cells in early apoptosis by between 20 and 25% starting from 24 h of incubation; however no further increase was seen at longer incubation times. Highest concentration of EtOAc crude extract (100 *μ*g/mL) produced maximal increase in percentage of cells in early apoptosis by 24 h. By 48 h, the percentage had dropped significantly to about 5%. A further significant decrease in percentage of cells in early apoptosis was seen at 72 h. Treatment of HepG2 cells with F7 also produced similar effects (Figure 3). A shorter incubation period of 24 h with F7 (12.5–37.5 *μ*g/mL) did not affect the cell population in early apoptosis. At 48 h, a concentration-dependent increase in percentage of cells in early apoptosis was seen. Highest concentration of F7 (37.5 *μ*g/mL) induced nearly 85% of cells to undergo apoptosis. This effect was inversely dependent on incubation time as percentage of cells undergoing early apoptosis decreased to approximately 50% after 72 h. With a longer period of incubation, HepG2 cells shifted from a situation of having maximum viability to entering early apoptosis and then to late-stage apoptosis and necrosis (Figure 4). Population of cells undergoing apoptosis upon exposure to F7 at its IC_50_, at 72 h, was smaller than at 48 h. Conversely, late apoptotic/necrotic cell population was higher at 72 h than at 48 h. These results show that at 72 h, F7 had induced necrotic cell death rather than apoptotic cell death. A range of stimuli such as radiation, hypoxia, heat, and cytotoxic compounds induce apoptosis at low concentrations but higher concentrations of these stimuli can result in necrosis [47]. Three types of cellular response to plant extracts have been proposed [48]. Mild exposure may cause mild oxidative stress that ignites cellular antioxidant defense systems; exposure to moderate to higher concentrations may gradually overwhelm antioxidant defense systems to induce apoptosis, while exposure to even higher concentrations for long periods may quickly overwhelm antioxidant defenses to stimulate prooxidative effects leading to cellular damage* via* necrosis. Two factors that transform an ongoing apoptotic process to a necrotic process are the availability of intracellular ATP and of caspases [49]. Extended exposure of cells to plant extract may cause loss of mitochondrial membrane potential and may promote release of* cytochrome-c* leading to distortion of electron transport and inhibition of Krebs cycle [50]. This will finally cause depletion in ATP and rapid generation of ROS which can both cause cell death without involvement of caspases [51]. Proapoptotic activity of apoptosome is dependent on availability of ATP; thus reduction of energy would cause a greater proportion of cells to undergo necrosis rather than apoptosis [52]. DNA fragmentation during apoptosis of HepG2 cells that were treated with F7 (12.5–37.5 *μ*g/mL) for 48 h was visualized* via* TUNEL assay using fluorescent confocal microscopy (Figure 5). Cells that were stained red were viable while bright green stains indicated apoptotic cell bodies with DNA fragmentation.

### 4.6. Expression of Genes Related to Apoptosis

**Figure 6:**
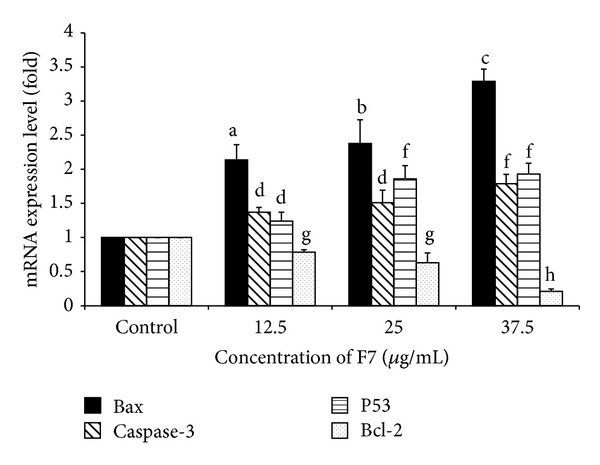
Effect of F7 on expression levels of apoptotic-related genes in HepG2 cells. HepG2 cells (1 × 10^6^/well) were incubated with F7 of* P. minus* (12.5–37.5 *μ*g/mL) for 48 h at 37°C. Results are mean ± S.D (*n* = 3). Values with different alphabets are significantly different (*P* < 0.05, ANOVA and Bonferroni test).

Molecular mechanism of apoptosis was investigated using RT-PCR by examining expression of key apoptotic-related genes. Primer sequences of the genes are shown in Table 6.* Bcl-2* gene is an antiapoptotic gene; overexpression of this gene would prevent apoptosis while* Bax* gene is a proapoptotic gene working oppositely.* P53* and* caspase-3* genes are vital in executing the apoptosis process in cells. Expression of* Bax*,* Bcl-2*,* p53*, and* caspase-3* genes in HepG2 cells that were treated with F7 (12.5–37.5 *μ*g/mL) for 48 h is shown in Figure 6. F7 induced a concentration-dependent increase in* Bax* expression. Compared to control untreated cell, mRNA expression of* Bax* was significantly elevated by about 3-fold at highest concentration of F7 (37.5 *μ*g/mL). The two lower concentrations of F7 produced about 2–2.5-fold increase in* Bax* expression. Similar concentration-dependent increase in expression levels of* caspase-3* and* p53* genes was seen in F7-treated cells. Lowest concentration of F7 significantly increased* caspase-3* expression from control. A significantly greater increase in* caspase-3* expression by about 1.8-fold was elicited by the highest concentration of F7 used. Expression of* p53* gene was significantly increased from control by lowest concentration of F7. Maximal increase in* p53* gene expression by about 2-fold was elicited by middle concentration of F7 (25 *μ*g/mL) as no further increase was seen at 37.5 *μ*g/mL. In contrast, expression of antiapoptotic gene,* Bcl-2*, was inhibited in concentration-dependent manner by F7. Highest concentration of F7 elicited maximal inhibition in* Bcl-*2 expression. Ratio of* Bax/Bcl-2* is crucial at determining susceptibility of cells to death signals [53]. Our results showed F7 induced apoptosis* via* upregulation of* Bax, p53*, and* caspase-3* genes and downregulation of* Bcl-2* gene. Activation of* p53* stimulates release of* cytochrome-c* from mitochondria [54]. Consequently, apoptosome complex is formed to further initiate effector caspases, including* caspase-3*, to execute the apoptosis process. Release of mitochondria apoptogenic factors is controlled by* Bax* and* Bcl-2* genes which either induced or suppress permeabilization of outer mitochondrial membrane [55]. Similar observations were reported in determination of antiproliferative effect of* Juglan regia* L. by assessment of apoptotic related genes [56].

### 4.7. Cell Cycle Arrest by F7

**Figure 7:**
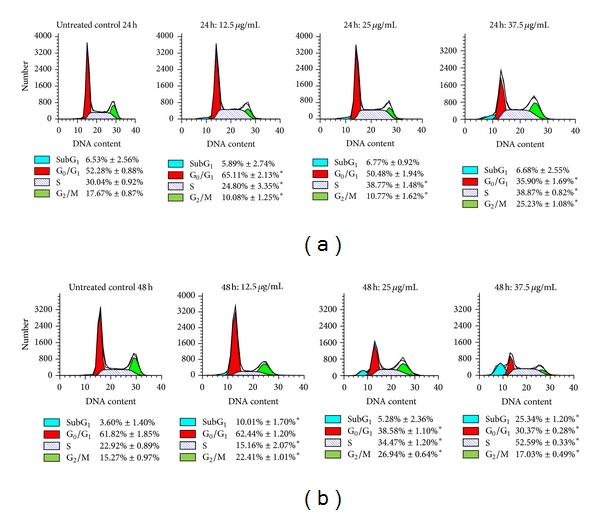
Effect of F7 of* P. minus* on cell cycle progression in HepG2 cells. Cells (1 × 10^6^/well) were treated with F7 (12.5–37.5 *μ*g/mL) for (top lane) 24 h or (bottom lane) 48 h at 37°C. Data are representative of three independent experiments. *Significantly different from untreated control cells (*P* < 0.05, ANOVA and Bonferroni test).

Treatment of HepG2 cells with F7 (12.5, 25, and 37.5 *μ*g/mL) for 24 and 48 h resulted in significant accumulation of cells at S-phase as compared to control untreated cells (Figure 7). The effect was concentration- and time-dependent. After 24 h of incubation with 25 or 37.5 *μ*g/mL F7, cell population at S-phase was increased compared to control while G_2_/M cell population was increased by the highest concentration of F7. This showed that F7 delayed cell progression through S-phase, earlier than G_2_/M phase, thus restricting cells from entering G_0_/G_1_ phase. Sub-G_1_ cell population after treatment with F7 at 24 h was not altered. Following 48 h of incubation, highest concentration of F7 increased Sub-G_1_ and S-phase populations to approximately 25.34 ± 1.20% and 52.59 ± 0.33%, respectively, while G_2_/M phase was decreased to 17.03 ± 0.49%. Sub-G_1_ phase is a hallmark of apoptosis [57]; therefore these findings are consistent with flow cytometry measurements of apoptosis in HepG2 cells treated with F7.* p53* also plays an important role in executing cell cycle arrest [58]. During S-phase, cellular DNA is replicated precisely and accurately to prevent genetic abnormalities which could lead to mutations or cell death [59] and* p53* is responsible for regulation of certain proteins which are related to cell cycle checkpoint.

## 5. Conclusion

In summary,* P. minus* as ethyl acetate crude extract or its fraction, F7, showed high antioxidant capacity and selective antiproliferative activity against HepG2 cells. The high antioxidant capacity of this herb is probably attributed to its polyphenolic content. Antiproliferative effect of F7 was directly correlated to its antioxidant capacity. Flow cytometry analyses and gene expression studies in F7-exposed HepG2 cells provided evidence that F7 induced apoptosis in time- and concentration-dependent manner through upregulation of proapoptotic genes and downregulation of antiapoptotic gene. F7 was antiproliferative towards HepG2 cells* via* inhibition of the cell cycle at S-phase. Continuing work to identify the compounds in F7 that are responsible for these activities is in progress in our laboratory. We are also looking at effects of F7 on a cancer model* in vivo*.

## Figures and Tables

**Table 1:** Antioxidant activities of four crude extracts of *P. minus*.

Crude extract	TPC (mg GAE/g extract)	DPPH scavenging activity (EC_50_) (*μ*g/mL)	FRAP value (*μ*mol Fe (II)/mg dry weight extract)
Pet. Ether	78.2 ± 3.2^a^	745.2 ± 20.2^a^	345.67 ± 4.9^a^
MeOH	128.2 ± 7.8^b^	540 ± 10.8^b^	781.32 ± 4.2^b^
EtOAc	227 ± 4.5^c^	120.3 ± 2.7^c^	2435.21 ± 28.9^c^
Water	132.3 ± 10.3^b^	472.5 ± 15.2^b^	842.61 ± 21.3^b^

Values are mean ± S.D (*n* = 3). Values with different alphabets in the same column are significantly different (*P* < 0.05, ANOVA and Bonferroni test). GAE: gallic acid equivalents.

**Table 2:** Antioxidant activities of fractions isolated from EtOAc crude extract of *P. minus*.

EtOAc fractions	TPC (mg GAE/g extract)	DPPH scavenging activity (EC_50_) (*μ*g/mL)	FRAP value *μ*mol Fe (II)/mg dry weight extract)
F1	ND	>1 mg/mL	ND
F2	14.2 ± 3.5^a^	568 ± 8.7^a^	147.53 ± 8.4^a^
F3	16.3 ± 4.2^a^	348.6 ± 5.7^b^	187.46 ± 10.7^b^
F4	21.7 ± 3.1^b^	200.2 ± 2.1^c^	224.41 ± 26.5^c^
F5	57.2 ± 6.8^c^	140.3 ± 3.2^d^	270.76 ± 17.2^d^
F6	96 ± 6.7^d^	90.2 ± 4.5^e^	356.84 ± 10.9^e^
F7	113.16 ± 6.2^e^	30.5 ± 3.2^f^	1169.1 ± 20.3^f^
Trolox	99 ± 2.4^d^	ND	ND
Chlorogenic acid	ND	19.95 ± 2.8^g^	ND
Ascorbic acid	ND	ND	3305.67 ± 51.5^g^

Values are mean ± S.D (*n* = 3). Values with different alphabets in the same column are significantly different (*P* < 0.05, ANOVA and Bonferroni test). GAE: gallic acid equivalents. ND: not determined.

**Table 3:** Qualitative analysis of phytochemicals of *P. minus*.

Phytochemical test	Extract of *P. minus *			
	Pet. ether	EtOAc	MeOH	Water
Flavonoids	−	+++	+	+
Alkaloids	−	+++	−	−
Saponins	++	++	−	+++

Remarks: +++: high intensity; ++: moderate intensity; +: less intensity; −: absent.

**Table 4:** IC_50_ values of *P. minus* extracts on different cell lines after 72 h of incubation.

Crude extract	HepG2	WRL 68	HeLA	HCT 116	MCF-7	Chang
	IC_50_ (*μ*g/mL)					
Pet. ether	>250^a^	56.23 ± 3.2^c^	127.21 ± 3.2^b^	145.32 ± 3.3^d^	>250^a^	>250^a^
EtOAc	32.25 ± 3.72^b^	122.38 ± 20.84^b^	63.09 ± 6.7^c^	199.52 ± 5.2^b^	>250^a^	285.72 ± 59.8^b^
MeOH	>250^a^	>250^a^	205 ± 4.3^d^	86.3 ± 3.5^c^	>250^a^	>250^a^
Water	>250^a^	>250^a^	>250^a^	>250^a^	>250^a^	>250^a^

HepG2 cells (2 × 10^6^/well) were incubated with crude extracts of *P. minus* for 72 h at 37°C. Results are mean ± S.D (*n* = 3). Values with different alphabets in the same column are significantly different (*P* < 0.05, ANOVA and Bonferroni test).

**Table 5:** IC_50_ values of different fractions from EtOAc extract of *P. minus* on HepG2 cell line.

Fractions	IC_50_ (*μ*g/mL)
F1	ND
F2	52.86 ± 2.4^a^
F3	63.19 ± 2.64^b^
F4	46.12 ± 6.1^a^
F5	65.96 ± 2.5^b^
F6	42.08 ± 3.22^a^
F7	25.75 ± 1.5^c^

HepG2 cells (2 × 10^6^/well) were incubated with *P. minus* fractions for 72 h at 37°C. Results are mean ± S.D. (*n* = 3). Values with different alphabets are significantly different (*P* < 0.05, ANOVA and Bonferroni test). ND: not determined.

**Table 6:** Gene name, base pair (b.p) size, and forward and reverse primer sequences that were used in the real time PCR experiment.

Gene name	b.p	Forward primer	Reverse primer
NM_031142.2 (*Actb*)	97	ATGGTGGGTATGGGTCAG	CAATGCCGTGTTCAATGG
NM_000633.2 (*Bcl2*)	177	CCACCAAGAAAGCAGGAAACC	GGCAGGATAGCAGCACAGG
NM_032991.2 (*Casp3*)	159	CTGGACTGTGGCATTGAGAC	ACAAAGCGACTGGATGAACC
NM_00141980.1 (*p53*)	113	AGAACGAGGAGACGGTAATAGTG	CAATGACCTGACTGATGGAACC
NM_004324.3 (*Bax*)	110	CAGATGTGGTCTATAATGC	CTAATCAAGTCAAGGTCAC
